# PCSK9/LDLR System and Rheumatoid Arthritis-Related Atherosclerosis

**DOI:** 10.3389/fcvm.2021.738764

**Published:** 2021-10-08

**Authors:** Aikaterini Arida, Aigli-Ioanna Legaki, Evrydiki Kravvariti, Athanasios Protogerou, Petros P. Sfikakis, Antonios Chatzigeorgiou

**Affiliations:** ^1^Joint Rheumatology Program, National and Kapodistrian University of Athens Medical School, Athens, Greece; ^2^Department of Physiology, Medical School, National and Kapodistrian University of Athens, Athens, Greece; ^3^Cardiovascular Prevention and Research Unit, Department of Pathophysiology, Medical School, National and Kapodistrian University of Athens, Athens, Greece; ^4^Institute for Clinical Chemistry and Laboratory Medicine, University Clinic Carl Gustav Carus, Technische Universität Dresden, Dresden, Germany

**Keywords:** rheumatoid arthritis, cardiovascular disease, inflammation, PCSK9, LDLR

## Abstract

**Background/Aims:** Rheumatoid arthritis (RA) is associated with the emergence of cardiovascular disease, while chronic inflammation is considered a common denominator for their parallel progression. The Proprotein convertase subtilisin/kexin type 9 (PCSK9)/LDL-Receptor (LDLR) system is of high importance during atherogenesis, via regulating the clearance of LDL from the circulation; nevertheless the role of this molecular mechanism during RA-related atheromatosis is not known.

**Methods:** Herein, high-resolution ultrasound measurements for arterial hypertrophy, atheromatosis and arterial stiffness as well as comprehensive biochemical profiling were performed in 85 RA patients. The circulating levels of PCSK9 and LDLR were measured and their potential associations as well as of the PCSK9/LDLR ratio with patients' characteristics and the degree of atherosclerosis were investigated.

**Results:** Increased LDLR levels and decreased PCSK9/LDLR ratio were found in RA patients with at least 2 atheromatic plaques as compared to the ones without any plaques. In addition the levels of both PCSK9 and LDLR were positively correlated with the presence of atheromatic plaques as an age- and gender- adjusted multivariate analysis revealed.

**Conclusions:** Our data imply that the PCSK9/LDLR system plays a significant role during RA-related atherosclerosis and may therefore be used as a screening tool for disease progression in the future.

## Introduction

Rheumatoid arthritis (RA) is a systemic inflammatory disease with considerable prevalence worldwide, characterized by arthritis mainly of the peripheral joints. RA associates with increased cardiovascular disease (CVD), which is a main cause of morbidity and mortality in these patients. While classical CVD risk factors—namely arterial hypertension, hyperlipidemia and insulin resistance—are of higher frequency and severity in these patients compared to the general population, they account for part of the accelerated CVD in RA (1–4). Thus, recent efforts have focused on the possible causal association between RA and CVD, based on the common background of chronic inflammatory processes and dysregulated immune response in RA and atheromatosis (5–7).

Impaired lipid metabolism, mainly due to familial forms of hyperlipidemia, is a major disorder leading to increased plasma levels of atherogenic low-density lipoprotein cholesterol (LDL-C) and consequently to accelerated atherosclerosis and CVD. To this end, LDL is a primary therapeutic target for reducing CVD risk (8, 9).

PCSK9 is a newly discovered protease that regulates the plasma levels of atherogenic LDL-C. Under physiological conditions, LDL particles are removed from the circulation by binding to the LDL receptor (LDLR) on the membrane of hepatic, endothelial and other cells and then internalized by endocytosis. The LDLR returns to the cell surface and binds to other LDL particles. PCSK9 binds to LDLRs at the surface of the cells, enhances their degradation and thus reduces the availability of LDLRs on the cell surface and LDL-C clearance. This ultimately leads to a significant increase in the plasma LDL-C levels. Therefore, in recent years, PCSK9 inhibition via monoclonal antibodies has become a new therapeutic option for patients unable to achieve LDL-C levels with other lipid-lowering drugs, such as statins (10–12).

The possible role of PCSK9 in the acceleration of CVD in RA has been a subject of interest in recent years, given that there is a bidirectional link between PCSK9 inhibition and inflammation (13, 14). However, data is very limited and contradictory, while a correlation between PCSK9 and subclinical CVD in RA is lacking. Furthermore, no studies have examined the relative association of the PCSK9/LDLR ratio and CVD. The aim of the present study was to examine whether PCSK9 or LDLR levels associate with markers of subclinical CVD in RA patients and whether PCSK9 levels or PCSK9/LDLR ratio can predict high CVD risk in RA.

## Materials and Methods

### Study Population and Subclinical CVD Markers

In this prospective study, we included consecutive RA patients who met the 1987 revised criteria of the American College of Rheumatology and attended Laikon Hospital's outpatient clinics (15). Patients were at least 18 years of age and did not have clinical CVD, Diabetes mellitus, malignancy, chronic renal failure, or other concomitant chronic or acute inflammatory disease. Moreover, participants must have had at least 3 years of disease duration, in order to study RA's impact on atherosclerosis. Patients receiving any lipid-lowering therapy were excluded from the analysis.

All RA patients were evaluated by high-resolution ultrasound for subclinical CVD, namely (a) arterial hypertrophy, (b) atheromatosis, and (c) arterial stiffness. Arterial hypertrophy of the common carotid arteries was assessed by intima-media thickness (IMT) (average of the maximal IMT from two measurements) measured adjacent to plaques if present. Atheromatosis was estimated by the presence of carotid and/or femoral artery plaques in the distal and proximal wall of eight arterial sites (left and right common, internal carotid arteries and carotid bulb, and common femoral arteries). Atheromatic plaques were defined as local increase of the intima media thickness (IMT) of >50% compared to the surrounding vessel wall, an IMT>1.5 mm or local thickening>0.5 mm. Finally, arterial stiffness was assessed by carotid to femoral pulse wave velocity (PWV) and pressure wave reflections by augmentation index (Aix@75) using pulse wave analysis methodology (Shygmocor, AtCor, Sydney, Australia) as previously described (3). Subjects abstained from food, drink, or any medication for 12 h prior to examination and all ultrasound measurements were performed by the same technician using high-resolution B-mode ultrasound (Vivid 7 Pro, GE Healthcare) with a 14-MHz multifrequency linear transducer.

The study was approved by the Institutional Body Review and all subjects provided informed consent according to the Declaration of Helsinki.

### PCSK9 and LDLR Measurement, Biochemical, and Clinical Parameters

Blood samples were collected from all patients prior to examination of vascular indices and the levels of PCSK9 and LDLR were measured in patients' plasma using ELISA kits from R&D systems (Minnesota, USA).

The presence of CVD risk factors, as well as biochemical parameters–including high sensitivity C-reactive protein (hs-CRP), total cholesterol (TC), HDL, LDL, triglycerides (TG)- were obtained from each patient's file. Measurements had to be within 3 months from examination. A reclassification of classical CVD risk factors was performed for hypertension by out-of-office blood pressure measurements (>130/80 mmHg by 7-day home BP monitoring or 24-h ambulatory BP monitoring) and hyperlipidemia by LDL plasma levels (>130 mg/dl). RA-related therapies were also recorded and RA disease activity was assessed by the Disease Activity Score on 28 joints (DAS28) (16).

### Statistics

Normality of sample distribution was examined by the Kolmogorov–Smirnov test. For description and variation of continuous variables, we calculated the means and standard deviations (SD) when sample had a normal distribution or the median and 25th and 75th percentile values when samples were not normally distributed. For estimating correlations of biochemical and vascular indices we used multiple regression after adjustment for age and gender. Stata version 12 (StataCorp, College Station, TX, USA) was used for analyses and *p* < 0.05 was considered as the level of statistical significance in all cases.

## Results

**Figure 1:**
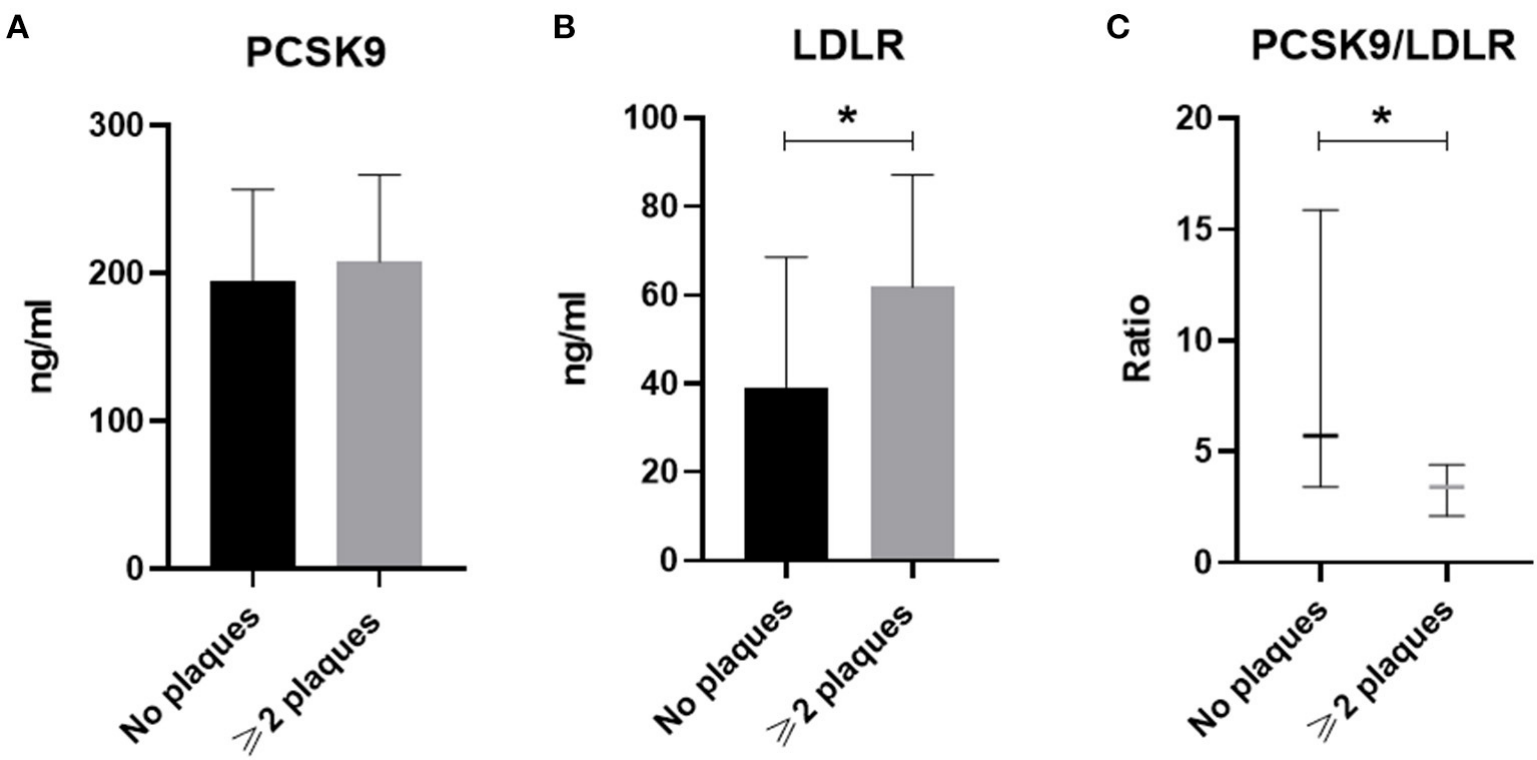
The circulating levels of PCSK9 (in **A**), LDLR (in **B**), and PCSK9/LDLR ratio (in **C**) in RA patients without plaques (*n* = 49) or with ≥2 plaques (*n* = 36) are shown. Data in **(A,B)** are presented as mean ± SD, while in **(C)** data are median and interquartile range of the 25th and 75th percentile of each group (*p < 0.05). PCSK9, Proprotein convertase subtilisin/kexin type 9; LDLR, low density lipoprotein receptor. *p < 0.05.

Overall, 85 RA patients (aged 59.2 ± 12.5, 15.3% men) fulfilled the inclusion criteria and their demographics, clinical and biochemical characteristics are presented in Table 1. Among them, 49 patients did not have atheromatic plaques (aged 53.1 ± 11.5, 12% men) and 36 had at least 2 atheromatic plaques (aged 67.4 ± 8.7, 19% men). Although no difference was found in the levels of PCSK9 between patients with no plaques as compared to those with at least 2 plaques, the latter were presented with increased LDLR levels and reduced PCSK9/LDLR ratio (Figure 1). Importantly, no difference at the levels of PCSK9, LDLR, or their ratio was found when RA patients were compared with age-, gender,- and atheromatosis status-matched healthy controls (data not shown), implying that the RA disease *per se* does not have an impact on PCSK9 and LDLR levels.

**Table 1:** Demographics, clinical characteristics, and subclinical vascular indices of RA patients.

		**RA patients (*n* = 85)**
Age (years)		59.2 ± 12.5
Gender (male) (%)		15.3
BMI (kgs/m^2^)		27.0 ± 4.8
DAS 28		2.45 ± 0.92
Biologic therapy (n)		36.5
C-reactive protein (mg/L)		3.2 (3.2–6.0)
Systolic BP (mmHg)		124.8 ± 18.5
Diastolic BP (mmHg)		74.4 ± 7.0
Map (mmHg)		87.4 ± 9.9
Smoking (%)	Never	44.7
	Current	21.2
	Ex	34.1
Hypertension (%) (after reclassification)^*^		40
Hyperlipidemia (%) (after reclassification)^**^		40.5
Antihypertensive drugs (%)		35.3
Lipid lowering drugs (%)		–
Plaques (%)	Carotid and/or femoral	42.4
	Carotid	36.5
	Femoral	35.3
IMT (mm)	RCCA	0.692 ± 0.133
	LCCA	0.741 ± 0.155
PWV (m/s)		8.45 ± 1.84
AIx@75 (%)		32.4 ± 10.6

* *YES if already treated or based on out-of-office BP measurements*.

** *YES if reported to be diagnosed in the past or (b) diagnosed if LDL measurement >130 mg/dl*.

*Data are mean ± SD*.

**Figure 2:**
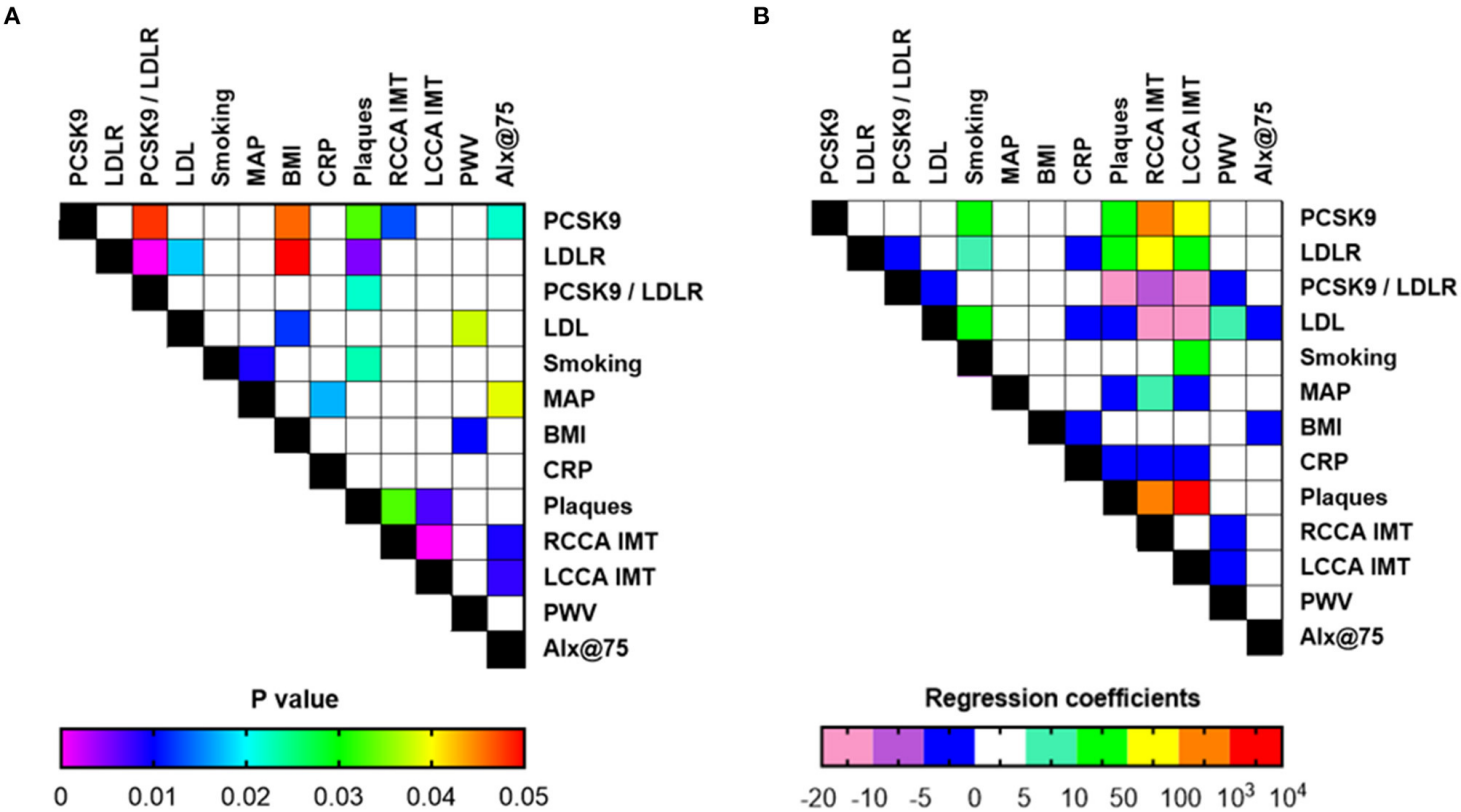
Associations of laboratory and clinical indices (**A**: *p*-values, **B**: coefficients or odds ratio for continuous and categorical variables, respectively) in all RA patients included in the study (*n* = 85) upon multiple regression analysis and adjustment for age and gender. PCSK9, Proprotein convertase subtilisin/kexin type 9; LDLR, low density lipoprotein receptor; MAP, mean arterial pressure; BMI, body mass index; CRP, C-reactive protein; RCCA, right common carotid artery; LCCA, left common carotid artery; IMT, intima-media thickness; PWV, pulse wave velocity; Alx@75, augmentation index.

Multivariate analysis, after adjusting for age and gender for all RA patients, revealed that PCSK9 was positively correlated with the presence of atheromatic plaques (*p* = 0.033), as well as IMT in the RCCA (*p* = 0.013) and a trend was observed for the LCCA (*p* = 0.060). Regarding arterial stiffness indices, PCSK9 was also associated with AIx@75 (*p* = 0.022), but not PWV. LDLR concentration was also associated with plaque presence by multivariate analysis (*p* = 0.005) and a trend was observed for IMT in the RCCA. No association was observed for any of the arterial stiffness indices under study (Figure 2 and Supplementary Table 1).

## Discussion

To our knowledge, this is the first study to examine the correlation of PCSK9, as well as LDLR, and markers of subclinical CVD, including PWV and AIx, in RA. Herein, we chose to exclude patients receiving lipid-lowering therapies, in order to attenuate any possible impact on PCSK9 concentrations. We found an association between PCSK9 plasma levels and atheromatic plaques, RCCA IMT, and AIx, even after adjustment for age and gender. Our findings are in accordance with current knowledge for the general population, where PCSK9 is associated with atherosclerosis and the progression of atheromatic plaques (17, 18). Moreover, LDLR levels were associated with increased plaque presence, while the PCSK9/LDLR ratio seems to work well as a screening tool for RA-related atheromatosis.

The association between PCSK9 and inflammation has been widely investigated in recent years. In RA, PCSK9 may also be implicated in the promotion of inflammation, contributing to the atherogenic background of the disease. In epidemiological studies, PCSK9 is positively correlated with inflammatory markers, such as CRP, white blood cell count and fibrinogenin patients with acute coronary syndromes (13). Additionally, PCSK9 has been found to activate the TLR4/NF-κB pathway and promote the secretion of pro-inflammatory cytokines in macrophages, induced by oxidized LDL (oxLDL) in a variety of tissues (13, 19, 20). It also affects the migration of monocytes, via regulation of the chemokine receptor CCR2, which plays a role in joint inflammation as well as in the development of atherosclerosis (21). Although PCSK9 inhibition does not seem to reduce C-reactive protein levels (22), PCSK9 silencing seems to directly decrease vascular inflammation in apolipoprotein E knockout mice (19).

Interestingly, PCSK9 promotes inflammation, which in turn may further stimulate the expression of PCSK9. In a recent study, LPS administration resulted in a marked increase in PCSK9 mRNA levels in the liver and the kidney, thus causing further increase to circulating LDL (23). Moreover, OxLDL has been found to upregulate the expression of PCSK9 macrophages in a dose-dependent manner, possibly by the secretion of inflammatory cytokines, such as IL-1, IL-6, and TNF-a (24).

Regarding the role of PCSK9 in RA, results from two studies are conflicting. Ferraz-Amaro et al. showed that RA patients had lower PCSK9 levels than healthy controls, after adjustment for classical CVD risk factors, lipids and statins; this was attributed to the “lipid paradox” seen in patients with RA (25, 26). However, patients included in this study had moderately active disease (DAS28 = 3.48) and even so, higher PCSK9 levels were associated with DAS28-CRP, but not CRP nor ESR, suggesting a link between inflammation and PCSK9. In our study, there was no correlation between CRP nor DAS28, however RA disease was in remission or low activity. On the other hand, in the study by Ferraz-Amaro et al. the difference in statin intake could have concealed even lower levels of PCSK9 in RA patients, as statins seem to increase PCSK9 concentrations and may explain the lack of association with LDL cholesterol (27). Importantly, PCSK9 was positively associated with cIMT and the presence of carotid plaques in patients with RA, however this association was lost after adjusting for classical cardiovascular risk factors. Oppositely, a small Chinese study reports that PCSK9 levels were significantly higher in RA patients vs. controls and also in patients with high disease activity vs. those with milder disease, indicating a link between PCSK9 and inflammatory status. Moreover, PCSK9 levels were also linked to lipid metabolism, in accordance with current knowledge (28).

While the role of PCSK9 in predicting CVD is well-established, at least in the general population, data on the predictive role of circulating LDLR is lacking; especially in the case of RA patients. LDLR is predominantly expressed by hepatocytes, as well as by endothelial cells, and circulating LDLR is considered a product of LDLR shedding from the cell surface. Importantly, previous studies have shown a strong correlation between the levels of circulating LDLR and those of LDL and triglycerides, implying that increased LDLR shedding is likely associated to impaired LDL clearance by the liver, thus favoring the emergence of atherosclerosis and CVD (29–31). In parallel, increased levels of PCSK9 may also result to decreased LDL clearance due to increased intracellular LDLR degradation (10). The absence of difference at the levels of PCSK9 in our case with a parallel increase of the levels of circulating LDLR, may indeed be associated with increased LDLR shedding in the presence of RA. Increased inflammation, such as that under RA conditions, may be the key factor via activation of sheddases, mostly those of the ADAM family, such as ADAM-17 (32). Further prospective studies specifically examining soluble LDLR are needed to elucidate the underlying mechanisms and pathophysiology.

Our study has few limitations. Firstly, the relatively small size of our cohort, which was unavoidable as patients under lipid-lowering therapy were excluded in order to reach more accurate results. Secondly, our RA patients were in remission or low disease activity; hence we could not evaluate an association between inflammation in RA, subclinical CVD and PCSK9 levels. Lastly, specific RA-related therapies were not included in the analysis, due to the small sample size, even if there are indications from recent publications that they could affect PCSK9 levels or even lipid profile in RA (33–35).

In conclusion, our data indicates that PCSK9 is associated with indices of subclinical CVD in RA. Nevertheless, whether there is a distinct pathogenic background or implication of disease-related inflammation or therapies affecting PCSK9 concentrations and acceleration of CVD in RA patients, needs to be examined in larger, prospective case-controls studies.

## Data Availability Statement

The raw data supporting the conclusions of this article will be made available by the authors, upon reasonable request.

## Ethics Statement

The studies involving human participants were reviewed and approved by National and Kapodistrian University of Athens Medical School. The patients/participants provided their written informed consent to participate in this study.

## Author Contributions

AA performed experimental work, literature search, wrote, and edited the manuscript. A-lL performed experimental work and edited the manuscript. EK interpreted data and edited the manuscript. AP interpreted data and edited the manuscript. PS designed the work, interpreted data, and edited the manuscript. AC designed the work, interpreted data, wrote and edited the manuscript. All authors have read and approved the final manuscript.

## Funding

The study was supported by grants from the European Foundation for the Study of Diabetes (EFSD) and the Hellenic Foundation for Research & Innovation (HFRI), both to AC.

## Conflict of Interest

The authors declare that the research was conducted in the absence of any commercial or financial relationships that could be construed as a potential conflict of interest.

## Publisher's Note

All claims expressed in this article are solely those of the authors and do not necessarily represent those of their affiliated organizations, or those of the publisher, the editors and the reviewers. Any product that may be evaluated in this article, or claim that may be made by its manufacturer, is not guaranteed or endorsed by the publisher.
