# Supplementary material for: PCSK9/LDLR System and Rheumatoid Arthritis-Related Atherosclerosis
Source: Front Cardiovasc Med. 2021 Oct 8;8:738764. doi: 10.3389/fcvm.2021.738764 (PMC8531404; doi:10.3389/fcvm.2021.738764)
Supplement: Supplementary file 1 [file Table_1.DOCX]

**Supplementary Material**

**Supplementary Table 1:** Associations of laboratory and clinical indices (A: p-values, B: coefficients or odds ratio for continuous and categorical variables respectively) in all RA patients included in the study (n=85) as presented in main Figure 2.

**A.**

| **PCSK9** | **LDLR** | **PCSK9 / LDLR** | **LDL** | **Smoking** | **Map** | **BMI** | **CRP** | **Plaques** | **RCCA IMT** | **LCCA IMT** | **PWV** | **AIx@75** |  |
| --- | --- | --- | --- | --- | --- | --- | --- | --- | --- | --- | --- | --- | --- |
| -- | 0.891 | 0.048 | 0.904 | 0.304 | 0.968 | 0.046 | 0.129 | 0.033 | 0.013 | 0.060 | 0.456 | 0.022 | **PCSK9** |
|  | -- | 0.000 | 0.018 | 0.429 | 0.292 | 0.05 | 0.494 | 0.005 | 0.070 | 0.318 | 0.863 | 0.384 | **LDLR** |
|  |  | -- | 0.240 | 0.990 | 0.466 | 0.929 | 0.226 | 0.022 | 0.673 | 0.232 | 0.348 | 0.921 | **PCSK9 / LDLR** |
|  |  |  | -- | 0.258 | 0.427 | 0.012 | 0.174 | 0.758 | 0.753 | 0.721 | 0.038 | 0.932 | **LDL** |
|  |  |  |  | -- | 0.009 | 0.465 | 0.686 | 0.023 | 0.915 | 0.14 | 0.488 | 0.234 | **Smoking** |
|  |  |  |  |  | -- | 0.080 | 0.017 | 0.115 | 0.503 | 0.878 | 0.118 | 0.039 | **Map** |
|  |  |  |  |  |  | -- | 0.29 | 0.967 | 0.663 | 0.488 | 0.010 | 0.371 | **BMI** |
|  |  |  |  |  |  |  | -- | 0.925 | 0.979 | 0.706 | 0.211 | 0.430 | **CRP** |
|  |  |  |  |  |  |  |  | -- | 0.033 | 0.007 | 0.522 | 0.374 | **Plaques** |
|  |  |  |  |  |  |  |  |  | -- | 0.000 | 0.888 | 0.009 | **RCCA IMT** |
|  |  |  |  |  |  |  |  |  |  | -- | 0.373 | 0.008 | **LCCA IMT** |
|  |  |  |  |  |  |  |  |  |  |  | -- | 0.125 | **PWV** |
|  |  |  |  |  |  |  |  |  |  |  |  | -- | **AIx@75** |

**B.**

| **PCSK9** | **LDLR** | **PCSK9 / LDLR** | **LDL** | **Smoking** | **Map** | **BMI** | **CRP** | **Plaques** | **RCCA IMT** | **LCCA IMT** | **PWV** | **AIx@75** |  |
| --- | --- | --- | --- | --- | --- | --- | --- | --- | --- | --- | --- | --- | --- |
| -- | 0.034 | 0.766 | 0.023 | 16.61 | 0.027 | 2.73 | 0.988 | 34.7 | 158.3 | 96.8 | 3.66 | 1.56 | **PCSK9** |
|  | -- | -0.883 | 0.23 | 6.88 | 0.377 | 1.38 | -0.239 | 23.6 | 60.5 | 29.9 | 0.469 | 0.318 | **LDLR** |
|  |  | -- | -0.074 | 0.073 | 0.166 | 0.04 | 0.07 | -10.18 | -7.09 | -18.4 | -1.17 | 0.021 | **PCSK9 / LDLR** |
|  |  |  | -- | 10.41 | 0.303 | 1.94 | -0.502 | -2.88 | -11.5 | -10.5 | 5.71 | -0.033 | **LDL** |
|  |  |  |  | -- | 0.915 | 0.959 | 0.988 | 3.58 | 0.807 | 12.6 | 0.899 | 1.033 | **Smoking** |
|  |  |  |  |  | -- | 0.395 | 0.255 | -3.98 | 6.64 | -1.23 | 1.18 | 0.22 | **Map** |
|  |  |  |  |  |  | -- | -0.054 | 0.055 | 2.24 | 2.87 | 0.985 | -0.05 | **BMI** |
|  |  |  |  |  |  |  | -- | -0.276 | -0.296 | -3.4 | 1.102 | 0.1 | **CRP** |
|  |  |  |  |  |  |  |  | -- | 721.62 | 8189.83 | 1.13 | 1.03 | **Plaques** |
|  |  |  |  |  |  |  |  |  | -- | 0.573 | -0.001 | 0.003 | **RCCA IMT** |
|  |  |  |  |  |  |  |  |  |  | -- | -0.009 | 0.004 | **LCCA IMT** |
|  |  |  |  |  |  |  |  |  |  |  | -- | 0.125 | **PWV** |
|  |  |  |  |  |  |  |  |  |  |  |  | -- | **AIx@75** |
